# A Compact Ultra-Thin 4 × 4 Multiple-Input Multiple-Output Antenna

**DOI:** 10.3390/s22166091

**Published:** 2022-08-15

**Authors:** Chuanba Zhang, Jianlin Huang, Xiaojing Shi, Guiting Dong, Jing Cai, Gui Liu

**Affiliations:** College of Electrical and Electronic Engineering, Wenzhou University, Wenzhou 325035, China

**Keywords:** 5G, MIMO, antenna pair, mobile phone, array

## Abstract

This article reported a compact ultra-thin tightly arranged 4 × 4 multiple-input multiple-output (MIMO) antenna pair (AP) functioning in the fifth-generation (5G) n78 band (3.4–3.6 GHz) for the ultra-thin 5G mobile handset. Two APs were printed on the center of two sideboards. A T-shaped open-ended slot was utilized in the grounding plane to improve the port impedance matching and attenuate the reciprocal magnetic coupling. A minimized total volume of 145 × 75 × 5 mm^3^ was obtained, and the area of each radiating unit was only 8.5 × 4.2 mm^2^ (0.1λ_0_ × 0.05λ_0_, λ_0_ is the free-space wavelength at the frequency of 3.5 GHz). By placing two radiating elements in an exceeding closed (1 mm or 0.01167λ_0_) distance, the designed AP precisely resonated at 3.5 GHz, and an acceptable measured isolation performance superior to 17 dB was attained. A prototype of this presented APs system was printed and tested, and remarkable consistency was observed between the simulated and measured curves. Numerous indicators were computed to assess its MIMO performance, such as Envelope Correlation Efficiency (ECC), Diversity Gain (DG), Total Active Reflection Coefficient (TARC), and Multiplexing Efficiency (ME).

## 1. Introduction

The tendency of rapid development of fifth-generation (5G) communication technology requires antennas with high data rates and low volume. Multiple-input multiple-output (MIMO) technology provides a promising solution to obtain a higher message transmission data rate than single-input single-output (SISO). Since the 3.5 GHz (3.3–3.8 GHz) band was initially deployed as one of the mobile phone spectrums, significant efforts have been made to exploit MIMO antennas operating at the 5G 3.3–3.8 GHz band for smartphones [1,2,3,4,5,6,7,8,9,10,11,12,13,14,15,16]. One thorny issue encountered in the antenna design is effectively accommodating as many antenna elements as possible in a space-restricted smartphone. In a MIMO system, the inevitable mutual magnetic coupling between any two radiating elements increases with decreasing distance. Numerous decoupling schemes have been presented to enhance antenna performance, such as the utilization of auxiliary decoupling branches [1,2,3,4], defected ground structure (DGS) [5,6,7,8], neutralization lines [9,10], lumped components [11,14], and orthogonal modes [12,13,14].

Recently, numerous MIMO smartphone antennas have been reported [12,13,14,15,16,17,18,19,20,21]. By integrating two antenna elements in a block and taking appropriate approaches to attenuate the mutual coupling, an antenna pair (AP) block was designed. The radiating elements of traditional smartphone antennas are were along two edges, and the APs system could reserve more space for 2G/3G/4G antennas integrated within a mobile handset due to its smaller dimension. A novel tightly arranged AP [12] was designed to construct a 4 × 4 MIMO system. By placing two antenna elements in an orthogonal mode in an AP block, a predominant isolation performance larger than 17 dB within 3.4–3.6 GHz was achieved. In ref. [15], a four-element self-decoupled AP system operating in 3.4–3.6 GHz was presented, and the mutual coupling in an AP block decreased to 17 dB by sharing one common grounding branch. A dual-band four-element AP system for 5G handsets was represented in ref. [16]. In ref. [17], a connecting line was inserted between two closed-located antenna elements in an AP block to weaken the mutual coupling in a wideband slot antenna. An acceptable isolation performance superior to 10.5 dB was maintained over the 3.3–5 GHz band.

Almost all lateral boards of the abovementioned APs systems exceed 6 mm, which is a great challenge to the realization of an ultra-thin smartphone. In ref. [18], a four-port MIMO AP system operating in the 5G n78 band (3.4–3.6 GHz) was studied and presented. Two APs were positioned in the center of the sideboard, and a decoupling (DC) stub was utilized further to improve the port impedance matching and isolation performance. Preferable isolation of around 17.5 dB between port 1 and port 2 was obtained in an AP block. The integral dimension of the proposed MIMO AP system was 150 × 75 × 6 mm^3^; 1 mm height reduction on the lateral board was realized compared with the aforementioned designs [12,15,16,17].

In this article, a minimized four-element AP system functioning in the 5G n78 band (3.4–3.6 GHz) for ultra-thin smartphones was provided. The proposed four-element MIMO antenna was made up of two APs, which were printed at the center of two sideboards. A T-shaped open-ended slot was utilized to attenuate the mutual coupling and improve the port impedance matching. The explored AP obtained a relatively small area of 18 mm × 4.2 mm, which is smaller than many reported APs [12,13,14,15,16,17,18,19,20]. The measured −10 dB bandwidth of the fabricated AP system is capable of covering the desired band, and ideal isolation performance superior to 17 dB was also maintained. Numerous indicators were computed to evaluate the MIMO performance, such as ECC, DG, TARC, and ME. Remarkable coherence between the simulated and measured curves was observed.

## 2. Antenna Design

**Figure 1:**
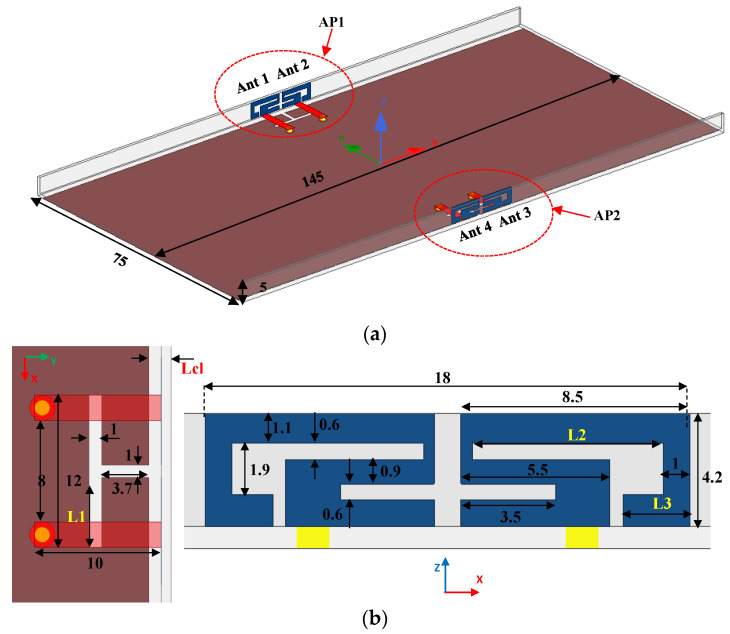
(**a**) The overall structure and (**b**) concrete construction of the designed AP system.

The general perspective of the four-port ultra-thin AP system is indicated in Figure 1. The overall size of the substrate FR4 with a loss tangent value of 0.02 and relative permittivity of 4.4 was 145 × 75 × 0.8 mm^3^. Two AP blocks were printed on the inner surface of the lateral board with a dimension of 145 × 4.2 × 0.8 mm^3^. The grounding plane with an area of 145 × 71.4 mm^2^ was printed on the bottom substrates. Two ground clearances (145 × 1.8 mm^2^) contribute to the impedance matching and resonant frequency adjustment. Each antenna element was excited through a 50 Ω microstrip line (2 × 10 mm^2^) and an SMA connector from the back of the design substrate. Figure 1b presents the detailed values of the proposed AP. The distance between two radiating elements was only 1 mm, which is shorter than the published AP systems [12,13,14,15,16,17,18,19,20]. Three crucial variables (*L1*, *L2*, and *L3*) affect antenna performance relatively distinctly and the width (*Lcl*) of the ground clearance, which is to be analyzed in a later section.

**Figure 2:**
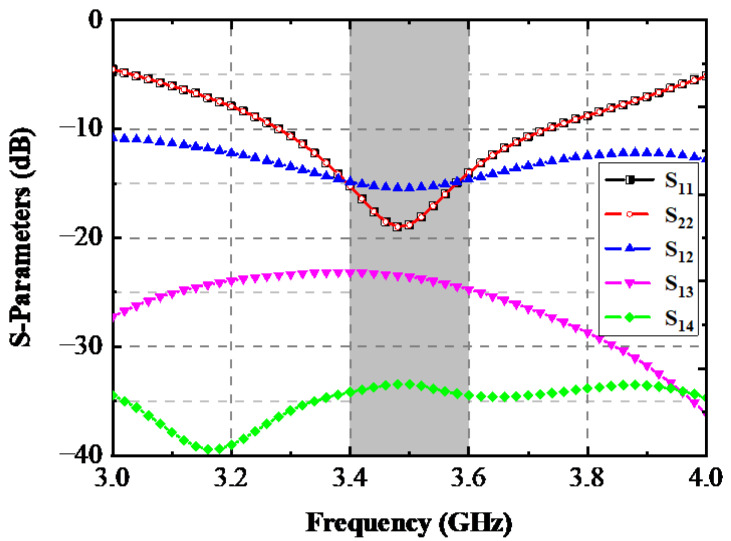
Simulated S-parameters.

The simulated S-parameters of this AP system are depicted in Figure 2. Since two AP blocks were placed symmetrically, only *S*_11_ and *S*_22_ emerged, and the transmission coefficients between Ant. 1 and Ant. 2, Ant. 1 and Ant. 3, and Ant. 1 and Ant. 4 are presented to evaluate the isolation performance. As displayed in Figure 2, the −10 dB impedance bandwidth of the AP was 0.45 GHz (3.31–3.76 GHz), which can entirely cover the target band of 3.4–3.6 GHz. Due to the introduction of a T-shaped open-ended slot on the ground plane, the mutual magnetic coupling between Ant. 1 and Ant. 2 was successfully enhanced to 15 dB within the desired band. In comparison, *S*_13_ and *S*_14_ were separately larger than 23 dB and 34 dB in the essential operating band.

## 3. Working Mechanism

### 3.1. Design Stages

**Figure 3:**
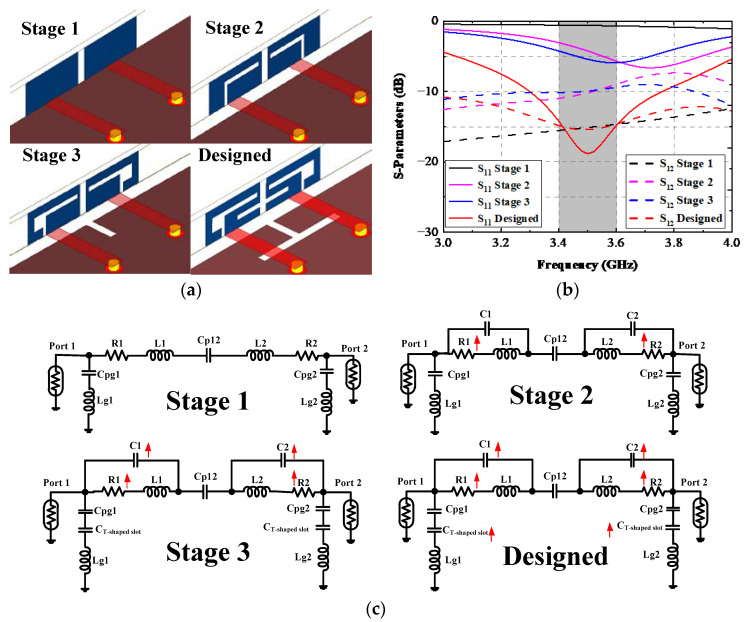
Simulated (**a**) design procedures, (**b**) simulated curves at each process, and (**c**) equivalent circuit of an AP at each stage.

The design stages of this antenna pairs system are discussed in this section to obtain a deeper comprehension of its mechanism. As plotted in Figure 3a, Stage 1 is a plain rectangle microstrip plane without ground clearance, and a resonant frequency larger than 4 GHz may exist, as depicted in Figure 3b. The circuit model of Stage 1 is a series branch of resistance and the inductance of the plain rectangle plane. The input impedance was relatively low because of the small resistance of the rectangular strip. By digging an L-shaped slit on the radiating element and cutting two ground clearances with a dimension of 145 × 1.8 mm^2^ on the grounding plane, Stage 2 was generated. A more apparent resonant mode around 3.7 GHz emerged; the resonant frequency shift is due to the effect of the parasitic capacitor brought by the introduced L-shaped slit. The utilization of the L-shaped slit greatly enhances the resistance of the radiating element. Hence, the port matching condition improved a lot. However, the isolation performance between Ant. 1 and Ant. 2 deteriorated a lot. To obtain a resonant frequency offset toward 3.5 GHz and isolation enhancement, two small rectangle slots with different areas were cut from the short edge of the L-shaped slit and the ground plane. One can see evidently from Figure 3b that the proposed AP resonated approximately at 3.55 GHz, and a slight improvement of about 3 dB of isolation performance was achieved in Stage 3. In the final designed structure, another rectangular slot was cut from the radiating elements, and the ground slot evolved into a T-shaped open-ended slot; the explored AP accurately resonated at 3.5 GHz, and the mutual coupling was also enlarged to 15.5 dB at 3.5 GHz. The equivalent circuit of an AP at each design step is illustrated in Figure 3c. R1(2) and L1(2) are the inherent resistance and inductance of Ant. 1(2) at each stage. Cpg1(2) and Lg1(2) are the parasitic capacitance between the radiating element 1(2) and the ground plane and the grounding inductance; Cp12 is the coupled capacitor between Ant. 1 and Ant. 2. C1(2) and C_T-shaped slot_ are parasitic capacitance brought by the slot cut from the antenna element and ground plane. The newly added component at each stage and the main variation are clearly indicated in Figure 3c.

**Figure 4:**
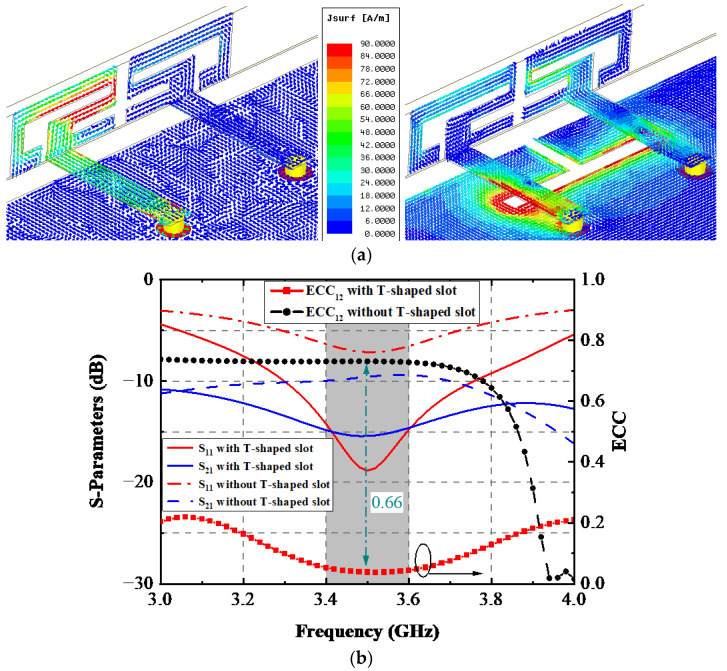
Simulated (**a**) surface current field and (**b**) results with the proposed AP without/with the T-shaped slot.

Figure 4a shows the simulated current distribution of the designed AP without/with the T-shaped slot when Ant. 1 is excited at 3.5 GHz. The most substantial surface current can be observed around the upper section of Ant. 1 when the T-shaped slot had not yet been applied. After the T-shaped slot was utilized, the most considerable current density was allocated over the edge of the T-shaped slot. A very weak current was obtained in the radiating element, thus decreasing the mutual coupling between two elements of the AP. The simulated outcomes of these two situations are plotted in Figure 4b. The exploited AP resonated at 3.5 GHz without a T-shaped slot, but the port impedance matching condition was not ideal enough. The −6 dB bandwidth range was 3.38–3.64 GHz, while the isolation performance was just around 9 dB within the desired bands. By introducing the T-shaped slot on the ground plane, a significant improvement in the port impedance matching condition was achieved; the −10 dB bandwidth was 3.31–3.76 GHz, and isolation between Ant. 1 and Ant. 2 was also lifted to 15 dB. It is quite noteworthy that a tremendous reduction of approximately 0.66 of ECC_12_ was attained after utilizing the T-shaped slot, which dramatically enhanced the MIMO performance of the designed AP.

### 3.2. Parameters Optimization

**Figure 5:**
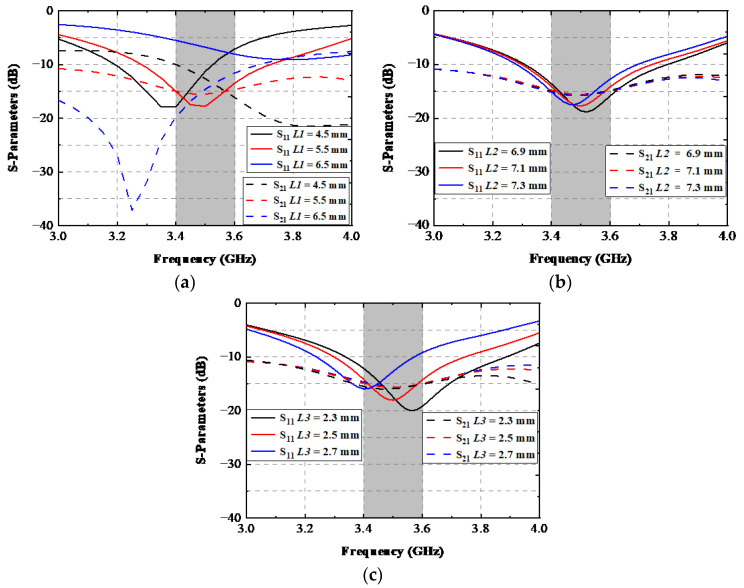
Simulated S-parameters with the various values of (**a**) *L1*, (**b**) *L2*, and (**c**) *L3*.

This section discusses four key parameters: *L1*, *L2*, *L3*, and *Lcl*. Variable *L1* mainly influences C_T-shaped slot_ shown in Figure 3c; *L2* primarily affects the resistance of R1(2); L3 principally affects C1(2) brought by the slot; *Lcl* affects both C_T-shaped slot_ and Lg1(2). As illustrated in Figure 5a, when the value of *L1* was 4.5 mm, the proposed AP resonated at approximately 3.4 GHz, and the isolation was more significant than 10 dB at 3.4 GHz. When *L1* was improved to 5.5 mm, the designed APs system precisely worked at 3.5 GHz, and the isolation was better than 15 dB at 3.5 GHz. As the value of *L1* was 6.5 mm, the −10 dB impedance matched band moved toward higher frequencies, a poor port impedance matching condition was observed, and the corresponding isolation performance deteriorated to 7 dB. Both parameters *L2* and *L3*, shown in Figure 5b,c, had the same impact on the port input return loss. With the increase of the value of *L2* or *L3*, the resonant frequency had a slight shift to lower frequencies while the isolation was almost unchanged. The optimized value of *L2* and *L3* was 7.1 mm and 2.5 mm, respectively.

**Figure 6:**
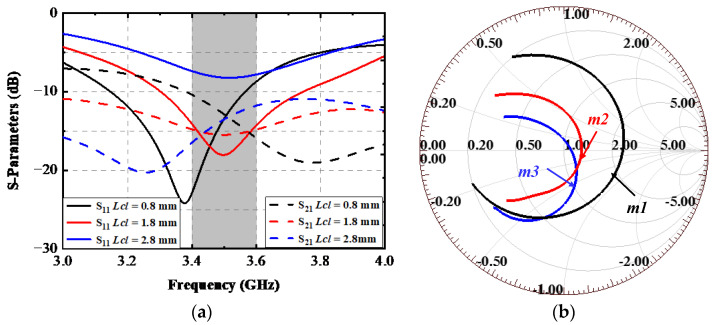
Simulated (**a**) S-parameters and (**b**) Smith chart with various *Lcl* values.

The impact resulting from the ground clearance is depicted in Figure 6. Figure 6a presents the simulated S-parameters as a function of *Lcl*, and the normalized impedance varying with the frequency of each situation is also illustrated in Figure 6b. The direction of clockwise indicates a frequency range of 3–4 GHz. When the value of *Lcl* was 0.8 mm, the simulated resonant frequency was 3.5 GHz, and the −6 dB bandwidth was 430 MHz (3.3–3.73 GHz). The impedance matching condition is not ideal enough. As shown in Figure 6b, the normalized impedance at 3.5 GHz when *Lcl* = 0.8 mm was 1.8393–0.7614 i (m1), which is far away from the center point of the Smith Chart; the mutual magnetic coupling between Ant. 1 and Ant. 2 was only larger than 10 dB within the interested band. When the value of *Lcl* was 1.8 mm, the designed AP still resonated at 3.5 GHz, but port impedance matching improved significantly. The −10 dB impedance-matched band is 3.31–3.76 GHz, which can contain the targeted band. The magnetic coupling between two ports in an AP is superior to 15 dB. Furthermore, the normalized impedance at m1, plotted in Figure 6b, was 1.256–0.1135 i (m2), which improved a lot compared with m3. When the value of *Lcl* is 2.8 mm; an apparent shift toward the left of the resonant frequency could be observed from Figure 6a, and the normalized port impedance at 3.5 GHz of this time is marked as m3 in Figure 6b, which is 1.1148–0.468 i.

### 3.3. Application Scenario

**Figure 7:**
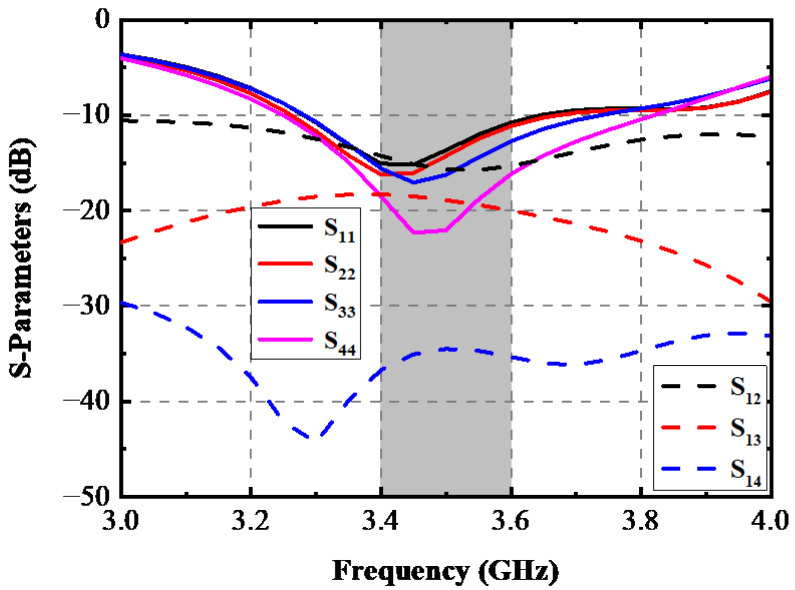
Simulated S-Parameters when this AP system is in SHM mode.

**Figure 8:**
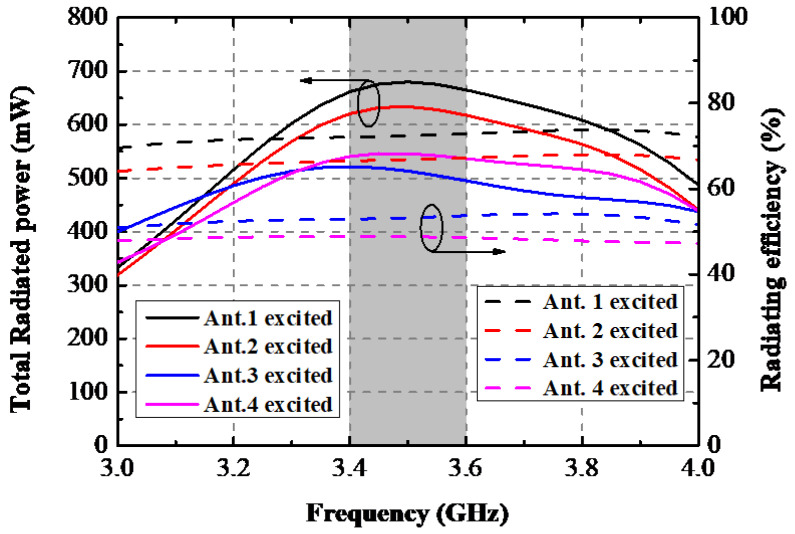
Simulated TRP and radiating efficiency of every antenna element.

**Figure 9:**
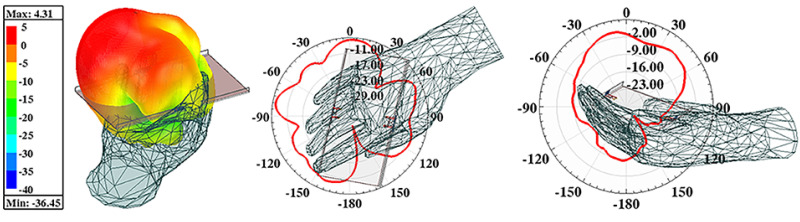
Simulated 3D and 2D radiating patterns of the proposed AP when Port 1 is excited at 3.5 GHz.

This paragraph presents a comprehensive analysis of the devices used in right-hand mode. Figure 7 depicts the simulated S-parameters when the proposed AP system held in a single right hand. A slight frequency offset caused by the inequable distance to the hand tissue of each element is observed, but the reflection coefficients of four ports can cover the desired bands. The isolation performance of this scene is also plotted in Figure 7. There was almost no significant deviation on *S*_12_ and *S*_14_ curves under this operation mode compared with Figure 2, while the deterioration of approximately 5 dB happened to *S*_13_ within the wanted band. Total Radiated Power (TRP) of the proposed AP system when a different port was excited with input power of 1 W at 3.5 GHz and radiating efficiency being portrayed in Figure 8. The TRP of Ant. 1 and Ant. 2 generally range from 600 mW to 700 mW in the target band, while Ant. 3 and Ant. 4 show poorer radiating ability due to their relative adjacent distance to the hand tissue. The hand tissue absorbs part of the input power. A relative flat radiating efficiency of the proposed AP was obtained. AP. 1 and AP. 2 achieved 70% and 50% radiating efficiency, respectively. The simulated three-dimension (3D) and two-dimension (2D) radiation patterns of this device held in a single hand are presented in Figure 9.

**Figure 10:**
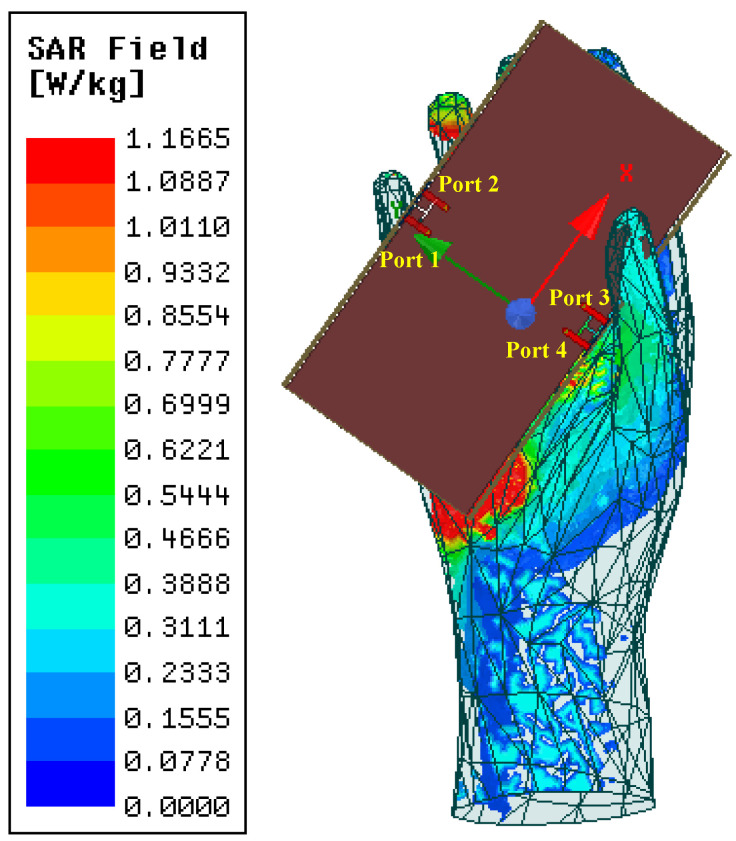
Simulated SAR field when Ant. 1 is excited at 3.5 GHz.

Specific absorption rate (SAR) is the definition of electromagnetic power absorption or consumption per unit of human tissue, which numerous electronics manufacturers have specified. Figure 10 displays the simulated SAR field of the hand model when Ant. 1 is excited with an input power of 100 mW at 3.5 GHz. It can be observed that the average SAR value of 1 Kg of human tissue was less than 1.166 W/Kg, which is inferior to the American and European standard values of 1.6 W/Kg and 2.0 W/kg.

## 4. Measured Results and Discussion

### 4.1. Measured Results

**Figure 11:**
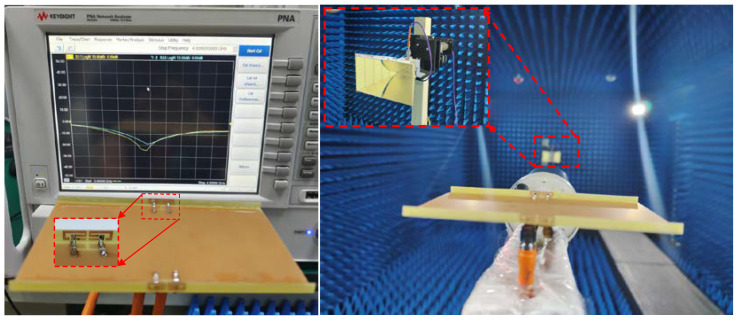
The fabricated model and experimental scene.

**Figure 12:**
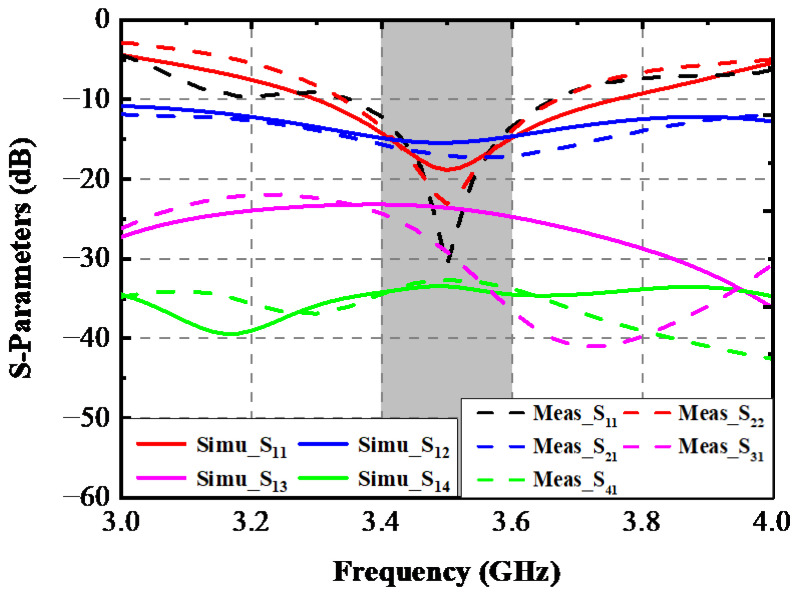
Measured and simulated S-parameters of the proposed AP system.

**Figure 13:**
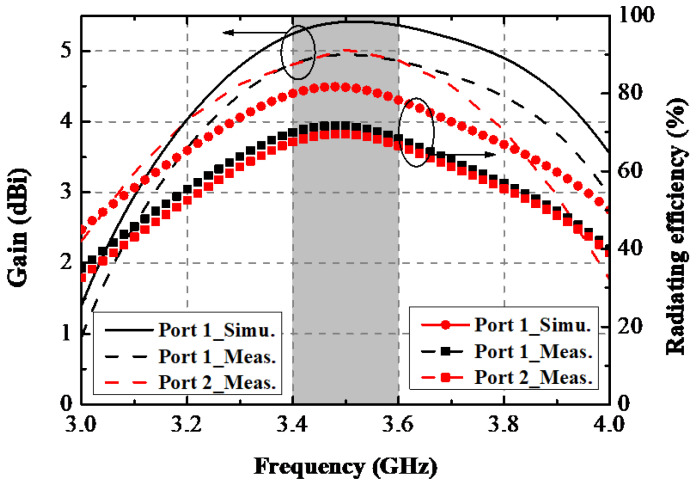
Measured and simulated gain and radiating efficiency of the proposed AP system.

A prototype of this designed antenna was manufactured. Figure 11 shows the printed model’s photograph and the testing scene by a Vector Network Analyzer (VNA: N5224A) and the anechoic chamber. In Figure 11, Ant. 1 and Ant. 2 are all excited; one can see that the proposed AP precisely resonated at 3.5 GHz, and the −10 dB impedance-matched band completely covered the target band. A slight difference between these two measured curves occurred due to the soldering procedure of the SMA connectors. Figure 12 makes a comparison between the simulated and measured S-parameters. The estimated −10 dB bandwidth was 290 MHz (3.36–3.65 GHz). The worst isolation performance of 17 dB appeared between Ant. 1 and Ant. 2. A general uniformity was achieved among the simulated and experimental curves. The measured gain and radiating efficiency when Ant. 1 and Ant. 2 were separately excited are portrayed in Figure 13. The maximum gain and peak radiating efficiency of the proposed AP within the target band were 5 dBi and 73%, respectively.

**Figure 14:**
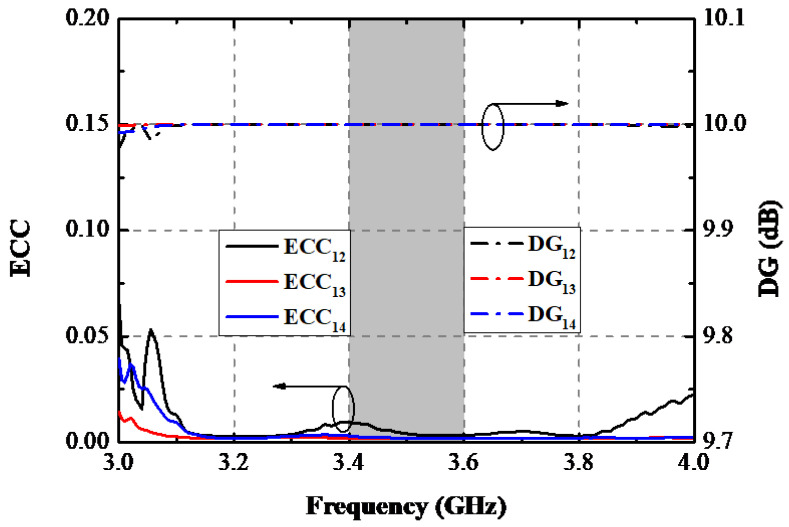
Measured ECCs and DGs.

ECCs, DGs, TARCs, and MEs were computed to assess the diversity performance of the proposed design. As a vital parameter to evaluate the MIMO performance of the MIMO system, ECC was calculated by Equation (1) to obtain a precise observation of the independent level of two antenna elements [19]. As illustrated in Figure 14, the worst ECC between Ant. 1 and Ant. 2 (Ant. 3 and Ant. 4) within the operational band was 0.012, while ECC_13_ and ECC_14_ were lower than 0.009 and 0.007, respectively. Figure 14 also presents the calculated DGs by formula (2) [18], and a 10 dB DG was realized in the target operating band, explaining that the reported AP is qualified to be applied to smartphones.

(1) $$ECC=\frac{{\left|S_{ii}^{*}S_{ij}+S_{ji}^{*}S_{jj}\right|}^{2}}{\left(1-{\left|S_{ii}\right|}^{2}-{\left|S_{ji}\right|}^{2})(1-{\left|S_{jj}\right|}^{2}-{\left|S_{ij}\right|}^{2}\right)}$$

(2) $$DG=10\times \sqrt{1-ECC^{2}}$$

**Figure 15:**
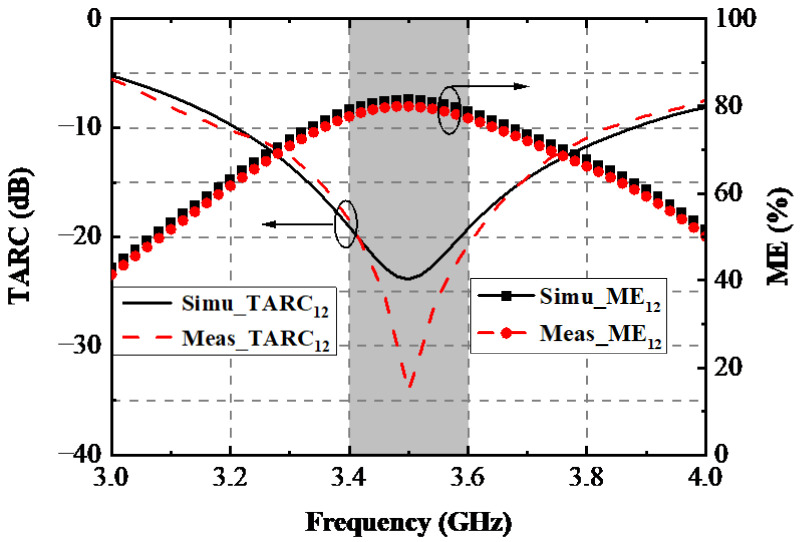
Computed TARC and ME.

**Figure 16:**
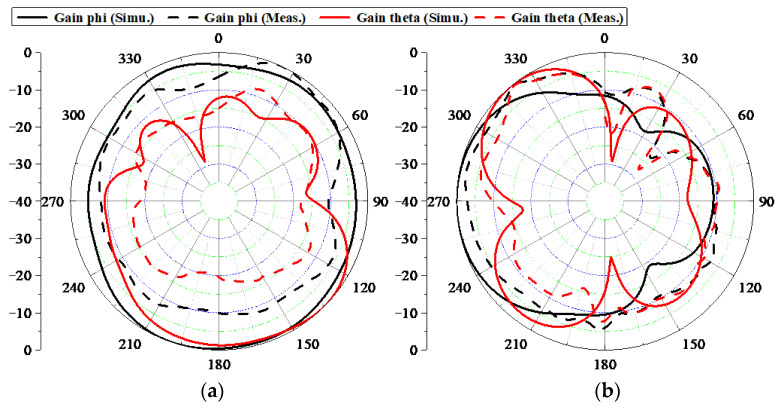
Measured 2D radiating pattern; (**a**) 3.5 GHz xoy plane. (**b**) 3.5 GHz xoz plane.

TARC is also a dominating indicator for analyzing the MIMO performance of a MIMO system, which can be calculated by the measured S-parameters using Equation (3) [22]. The computed TARC curves are shown in Figure 15. An admirable TARC of less than −10 dB during the desired band was achieved. ME is the definition of the power loss of a realistic antenna in attaining a given power capacity, compared with an ideal antenna with one hundred percent radiating efficiency and zero correlation between antenna elements. ME can be expressed [23] under the assumption of high SNR. The calculated MEs are depicted in Figure 15. Figure 16 displays the measured 2D radiating patterns of the proposed AP when Ant. 1 and Ant. 2 are separately excited.

(3) $$TARC=\sqrt{\frac{{\left(S_{11}+S_{12}\right)}^{2}+{\left(S_{22}+S_{21}\right)}^{2}}{2}}$$

(4) $$ME=\sqrt{\eta_{1}\eta_{2}(1-ECC_{12}^{2})}$$

Table 1 compares the designed device and other reported similar MIMO AP systems for the 5G n78 band. One can see from the table that the primary highlights of our work are the lower side board height (5 mm), shorter distance (1 mm) between two elements of the AP, and smaller area of the AP. Otherwise, a simple T-shaped slot on the ground plane effectively promotes the device’s MIMO performance. The measured outcomes showed that the proposed APs system can yet be regarded as an outstanding contender for ultra-thin smartphones.

### 4.2. Discussion

**Figure 17:**
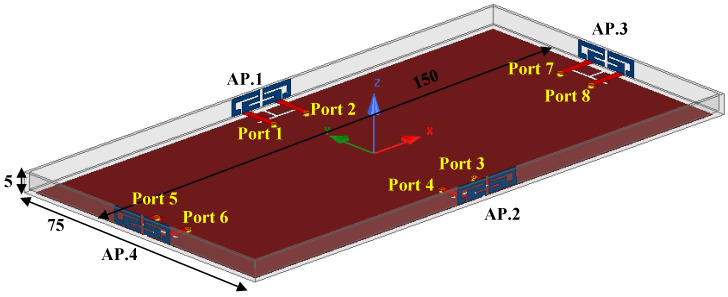
The overall structure of the discussed eight-port AP system.

**Figure 18:**
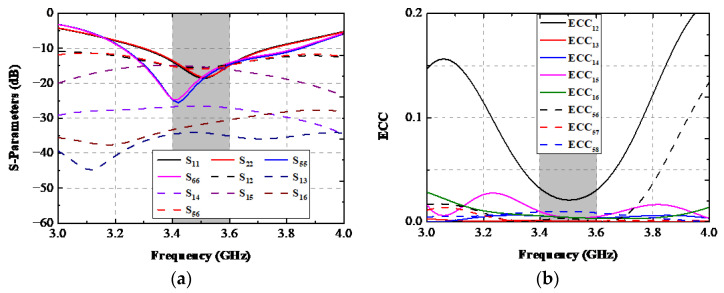
Simulated (**a**) S-parameters and (**b**) ECCs of the 8-port APs system.

An eight-port antenna structure is discussed in this paragraph. As presented in Figure 17, two shorter lateral frame boards were further constructed along two sharp edges of the system board described in Figure 1. Two ground clearances with a size of 71.4 × 1.8 mm^2^ were cut to obtain a uniform resonating condition as the other two existing APs. The simulated S-parameters of the eight-port AP structure are illustrated in Figure 18a. The −10 dB bandwidth of two existing APs still can cover the target band, and there was almost no deterioration in isolation performance compared with before. However, a resonating frequency shift of about 100 MHz occurred to AP.3 and AP.4, and the −10 dB operating band of AP.3 and AP.4 was 3.23–3.82 GHz, which can also wholly contain the desired band. A remarkable coherence between *S*_12_ and *S*_56_ was achieved, and the isolation performance between any two ports is still superior to the 15 dB level. The simulated ECCs of the discussed eight-port AP system is shown in Figure 18b. When Ant. 1 was excited at 3.5 GHz, the worst ECC within the essential band appeared in ECC_12_ (0.035).

## 5. Conclusions

A minimized four-port MIMO APs system operating in the 5G n78 band was studied for future ultra-thin smartphones. The designed AP obtains the advantages of low area (18 × 4.2 mm), closed fabricated distance (1 mm), and high isolation (17 dB). Numerous vital parameter analyses were performed to deeply comprehend this device. An application scene used in single-hand mode analysis is also provided to better understand its robustness characteristics and practicability. The explored antenna was printed and measured. The measured −10 dB bandwidth and worst isolation of the designed AP were 290 MHz (3.34–3.65 GHz) and 17 dB (*S*_21_), respectively. Lower ECC (0.009), preferable gain (5 dB), and high radiating efficiency (73%) were obtained. The last section also further discusses a four-AP eight-port structure. The explored MIMO APs system is expected to be utilized in future ultra-thin smartphones.

## Figures and Tables

**Table 1:** Performance contrast between the proposed AP system with other state-of-arts.

Design	Operating Band (GHz)	Total Size (mm^3^)	Dimension of an AP (mm^3^)	Port Distance in an AP (mm)	Isolation (dB)	ECC
[12]	3.4–3.6 (−10 dB)	150 × 73 × 7	12 × 7 × 0.8		17	0.07
[15]	3.4–3.6 (−10 dB)	150 × 73 × 7	20 × 6.2 × 0.8	15.6	17	0.1
[18]	3.4–3.6 (−10 dB)	150 × 75 ×6	23.5 × 5.2 × 0.8	11.7	17.5	0.009
[19]	3.4–3.6 (−10 dB)	150 × 75 × 6	30 × 5.2 × 0.8	7	16.5	0.03
This work	3.34–3.65 (−10 dB)	145 × 75 × 5	18 × 4.2	8	17	0.009

## Data Availability

Not applicable.
